# *Mycoplasma pneumoniae* PCR positivity rate among children in Chengdu, China: a retrospective cross-sectional epidemiological study

**DOI:** 10.3389/fped.2026.1903122

**Published:** 2026-10-02

**Authors:** Zhiyong Liao, Xiaomei Zou, Jing Liang, Meimei Lai, Qin Wang, Shuzhe Yang, Yao Deng, Chenggui Liu, Weijun He

**Affiliations:** 1 Department of Clinical Laboratory, Chengdu Women’s and Children’s Central Hospital, School of Medicine, University of Electronic Science and Technology of China, Chengdu, China; 2 Department of Clinical Laboratory, Simaqiao Caojiaxiang Community Health Service Center, Chengdu, Sichuan, China; 3 Department of Performance appraisal management, Chengdu Women’s and Children’s Central Hospital, School of Medicine, University of Electronic Science and Technology of China, Chengdu, China; 4 Department of Clinical Laboratory, Sichuan Province Orthopedic Hospital, Chengdu, China

**Keywords:** children, epidemiological characteristics, Mycoplasma pneumoniae, polymerase chain reaction, positivity rate, respiratory tract infections

## Abstract

**Background:**

*Mycoplasma pneumoniae* (MP) is one of the most common pathogens responsible for respiratory infections in children. The objective of this study was to investigate MP positivity rate and its distribution characteristics across different clinical diagnostic groups among children in Chengdu, China.

**Methods:**

A retrospective cross-sectional study was conducted at a single center in Chengdu, China, involving children aged 0–18 years who underwent MP polymerase chain reaction (PCR) testing based on clinician requisition or self-requested laboratory testing (SRLT) between January 1 and December 31, 2024. *χ*2 tests and multivariable logistic regression models were applied for statistical analyses.

**Results:**

Among 94,889 children (51,105 males and 43,784 females), the overall MP positivity rate was 12.3%, with no significant difference between sexes. Across five clinical diagnostic groups, MP positivity rate was highest in the acute lower respiratory tract infections (ALRTIs) group (13.9%), followed by the SRLT group (12.8%); both were significantly higher than those in the chronic respiratory diseases (CRDs) group (8.8%), the acute upper respiratory tract infections (AURTIs) group (6.6%), and the non-respiratory diseases (NRDs) group (4.8%). MP positivity rates in the ALRTIs, AURTIs, NRDs, and SRLT groups showed significant seasonal variation, peaking in winter, followed by spring; both seasons exhibited significantly higher positivity rates than summer and autumn. Monthly MP positivity rate peaked in January and February across all groups, after which it generally decreased with mild fluctuations, with the ALRTIs group consistently exhibiting the highest positivity rate. Among children with ALRTIs and AURTIs, MP positivity rates were significantly higher in school-aged, preschool, and adolescent children than in toddlers, infants, and newborns (*p* < 0.001). Multivariable logistic regression analysis revealed that MP positivity was independently associated with healthcare setting (inpatient vs. outpatient/emergency, OR: 1.24, 95% CI: 1.15–1.35), and PCR assay type (multiplex vs. singleplex, OR: 2.45, 95% CI: 2.25–2.67).

**Conclusions:**

Among children in Chengdu in 2024, MP PCR positivity peaked in winter (January–February) and was highest in school-aged children. These single-year findings may help inform local surveillance and guide future long-term epidemiological monitoring.

## Introduction

*Mycoplasma pneumoniae* (MP) is one of the smallest self-replicating bacterial pathogens. As a prokaryotic organism lacking a rigid cell wall, it is capable of independent survival—with its adhesion to host cells positively correlated with virulence—and differs fundamentally from both typical bacteria and viral pathogens, while its lack of a rigid cell wall is a core structural trait determining its clinical performance and laboratory detection characteristics (1, 2). This cell wall-specific property also leads to unique pharmacological susceptibility and staining features that distinguish MP from conventional bacterial pathogens (3, 4).

MP stands as one of the most prevalent pathogens responsible for respiratory tract infections (RTIs) in clinical settings, with a particularly notable impact on pediatric populations (5, 6). As a major causative agent of community-acquired pneumonia (CAP), MP infection in children presents a wide range of clinical manifestations: from mild acute upper respiratory tract infections (AURTIs) to severe acute lower respiratory tract infections (ALRTIs), along with the potential to induce both acute upper and lower respiratory tract inflammation and extrapulmonary syndromes (7, 8). MP is endemic worldwide, with epidemics occurring every few years. It is a major pathogen causing community-acquired respiratory infections in school-age children, accounting for 10%–40% of CAP cases among inpatients (9, 10).

A widespread post-COVID-19 resurgence of MP infections swept across Europe and Asia starting in the summer of 2023, accompanied by marked increases in MP-related hospitalizations in several European countries. The ratio of intensive care unit (ICU) admissions in the 2023–2024 season vs. previous seasons exceeded two in France, the United Kingdom, and the Netherlands, with adults also appearing to be at increased risk for severe disease compared with pre-pandemic outbreaks (11). During the 2023–2024 nationwide MP epidemic in France, the proportion of patients developing severe disease requiring intensive care reached 32.4% (12). In Guangzhou, China, the MP positivity rate among inpatients with acute respiratory tract infections (ARTIs) reached 29.4% in 2023, and children with severe MP pneumonia had more complications (13). Beyond respiratory tract involvement, MP infection may even be associated with exacerbations of chronic respiratory diseases (CRDs) or non-respiratory diseases (NRDs) in some cases (14–16).

In China, pediatric MP infection has long been a major public health concern affecting children's health, with studies reporting peak infection rates in school-age groups and obvious seasonal variations (17, 18). Core laboratory diagnostic approaches for MP infection mainly include culture, serological antibody testing, and nucleic acid amplification tests (NAATs) (19). *The Expert Consensus on the Diagnosis and Treatment of Mycoplasma Pneumoniae Pneumonia in Children (2023 Edition)* summarizes the characteristics of each diagnostic method: culture is regarded as the diagnostic gold standard but is rarely applied clinically due to cumbersome operation, long incubation cycle and low positivity rate; serological diagnosis relies mainly on changes in antibody titers; NAATs such as polymerase chain reaction (PCR) and simultaneous amplification and testing (SAT) are the preferred assays for identifying active MP infection owing to high sensitivity and specificity (20).

In recent years, with changes in climate, population mobility, and antibiotic use patterns, the epidemiological characteristics of MP infection—including age-specific positivity rates, seasonal distribution, and clinical manifestation spectrum—have shown regional variations, highlighting the need for localized epidemiological investigations (21, 22). Against this background, this study aimed to systematically analyze the epidemiological characteristics of MP infection in children in Chengdu by collecting data on children aged 0–18 who underwent MP testing (via singleplex or multiplex PCR for detecting MP in respiratory specimens) in local medical institutions. Specifically, the study investigated MP positivity rate (i.e., MP detection rate), its distribution across different age groups, seasons, and clinical diagnostic types, as well as its association with healthcare settings (inpatient vs. outpatient/emergency) and sex (males vs. females). The results are expected to provide updated and localized evidence for guiding clinical practice and public health decision-making related to pediatric MP infection in Chengdu and similar subtropical regions in southwestern China.

## Materials and methods

### Study design

This retrospective cross-sectional study was reported in accordance with the Strengthening the Reporting of Observational Studies in Epidemiology (STROBE) guidelines for cross-sectional studies. The research was conducted from January 1, 2024, to December 31, 2024, at Chengdu Women's and Children's Central Hospital, School of Medicine, University of Electronic Science and Technology of China, a Grade-A tertiary hospital for women and children with an annual outpatient volume of over 2 million visits. The hospital primarily delivers pediatric, obstetric, and gynecological care to residents across Chengdu and adjacent areas in southwest China. It has a well-defined, stable catchment population and receives referrals from urban and suburban areas within this region.

### Study participants

Pediatric patients aged 0–18 years receiving MP PCR testing at our institution were included. All tests were ordered either at the clinician’s discretion for suspected MP infection or via self-requested laboratory testing (SRLT) without prior physician consultation. Pharyngeal swabs were collected for MP detection, and two clinical PCR platforms were used: singleplex PCR and multiplex PCR. Testing modalities (singleplex vs. multiplex PCR) were assigned according to clinicians’ orders or SRLT applications.

Exclusion criteria were as follows: (a) participants with incomplete basic information—specifically, those missing clinical diagnosis records (for clinician-ordered tests only; SRLT participants were exempt from this requirement), or missing age or sex data; (b) those who underwent repeated testing within 28 days after the initial test; and (c) individuals over 18 years of age. Participants were categorized into six age groups: newborns (0–28 days), infants (29 days–1 year), toddlers (1–3 years), preschool children (3–6 years), school-aged children (6–12 years), and adolescents (12–18 years).

According to clinical diagnosis, participants were categorized into five groups: ALRTIs, AURTIs, CRDs, NRDs, and SRLT. For participants with multiple diagnoses, the first listed diagnosis was used for classification in this study.

1. The ALRTIs group comprised pneumonia, acute tracheitis, acute bronchitis, acute tracheobronchitis, acute laryngotracheitis, acute laryngotracheobronchitis, and acute bronchiolitis.
2. The AURTIs group comprised acute pharyngitis, acute laryngitis, acute tonsillitis, acute rhinitis, acute sinusitis, acute epiglottitis, and the common cold.
3. The CRDs group comprised chronic pharyngitis, chronic rhinitis, chronic sinusitis, chronic bronchitis, bronchiectasis, bronchial asthma, and pulmonary tuberculosis.
4. The NRDs group comprised Henoch-Schönlein purpura (allergic purpura), thrombocytopenic purpura, leukemia, hemophilia, infectious mononucleosis, hypothyroidism, hyperthyroidism, type 1 diabetes, diarrhea, acute gastroenteritis, acute appendicitis, acute parotitis, acute lymphadenitis, acute urinary tract infection, sepsis, epilepsy, myocarditis, Kawasaki disease, acute conjunctivitis, neonatal hyperbilirubinemia, acute urticaria, constipation, and other NRD diseases.
5. The SRLT groupcomprised cases of self-requested MP testing, defined as those in which the laboratory requisition was generated by the participant or their legal guardian via a self-service kiosk without prior physician consultation or clinical assessment.

### Specimen collection

Pharyngeal swab specimens were collected by clinicians or nurses who completed standardized training and passed assessment, in strict accordance with a unified age-adapted standard operating procedure (SOP). For inpatients, sampling was performed by ward clinicians or nurses; for outpatient and emergency patients, dedicated nurses from the Sampling Department undertook specimen collection. All collected samples were transported to the laboratory within 24 h by professionally trained and assessed transport staff of the Specimen Transportation Department.

### Detection of MP DNA via singleplex and multiplex real-time PCR

MP nucleic acid testing was performed using two commercial real-time PCR kits following the manufacturers’ standard protocols.

#### Singleplex PCR

A singleplex MP real-time PCR kit (Tianlong Science and Technology Co., Ltd., Suzhou, China) was run on a Gentier 96E Real-Time PCR System (Tianlong Science and Technology Co., Ltd., Xi’an, China), with a limit of detection (LOD) of 500 copies/mL for MP.

#### Multiplex PCR

A multiplex respiratory pathogen PCR kit (Sansure Biotech Inc., Changsha, China), which enables simultaneous detection of MP and five other respiratory pathogens, was run on an MA-6000 Real-Time Quantitative Thermal Cycler (Suzhou Molarray Co., Ltd., Suzhou, China). According to the manufacturer's specifications, the MP LOD of this multiplex assay was also 500 copies/mL.

All laboratory technicians were blinded to patients’ clinical diagnoses during nucleic acid testing and Ct value interpretation. Rigorous quality control measures specified by each kit's instructions were implemented in every testing batch: positive, negative, and internal amplification controls were tested alongside clinical specimens to monitor extraction efficiency, amplification success, and potential contamination. Per the manufacturers’ validation data, neither kit cross-reacts with other common respiratory pathogens, and both assays demonstrate high precision with a coefficient of variation (CV) of Ct values ≤5%. Both PCR systems were clinically validated for pediatric pharyngeal swab samples. Consistent MP positivity results were observed in routine clinical testing across the two platforms, suggesting acceptable analytical comparability and agreement between singleplex and multiplex MP detection.

## Statistical analysis

Data analysis was performed using SPSS (Statistical Package for Social Science) version 19.0 (SPSS Inc., Chicago, IL, USA). MP positivity rate was reported as percentages, and the Chi-Square test (*χ*^2^) was applied to perform global intergroup comparisons. For pairwise *post-hoc* comparisons between multiple subgroups after the overall Chi-Square test, Bonferroni correction was adopted to adjust *p*-values for multiple testing. 95% Wilson confidence intervals were calculated for monthly and annual MP positivity rates within each diagnostic subgroup. To systematically explore factors associated with MP positivity, univariate logistic regression was first performed for each candidate variable. Subsequently, multivariable logistic regression analysis using the Stepwise Forward Wald method was conducted. Six candidate variables—healthcare setting, sex, age group, season, clinical diagnosis, and PCR assay type (singleplex vs. multiplex)—were entered as candidates into the Stepwise Forward Wald model. Variables were retained if *p* < 0.05 and removed if *p* > 0.10. Sex was not retained in the final multivariable model. Adjusted odds ratios (OR) and their 95% confidence intervals (95% CI) were computed to quantify the strength and direction of associations. All statistical hypothesis tests were two-sided, and a *p*-value <0.05 was considered statistically significant.

## Results

### Study population characteristics

A total of 94,889 eligible children were included in this study, comprising 51,105 males and 43,784 females. Based on the healthcare setting, participants were classified into outpatient/emergency children (*n* = 62,429) and inpatients (*n* = 32,460). Testing modalities were assigned as follows: 55,699 children (29,258 males, 26,441 females) underwent singleplex PCR testing, and the remaining 39,190 children (21,847 males, 17,343 females) received multiplex PCR testing. The participant screening flowchart stratified by singleplex and multiplex PCR for eligible children aged 0–18 years is presented in Figure 1**.** Participants were categorized into six age groups: 1,469 newborns (0–28 days), 9,426 infants (29 days–1 year), 17,801 toddlers (1–3 years), 35,738 preschool children (3–6 years), 27,912 school-aged children (6–12 years), and 2,543 adolescents (12–18 years). According to clinical diagnosis, participants were categorized into five groups: ALRTIs (*n* = 72,752), AURTIs (*n* = 12,601), CRDs (*n* = 363), NRDs (*n* = 5,466), and SRLT (*n* = 3,707).

**Figure 1:**
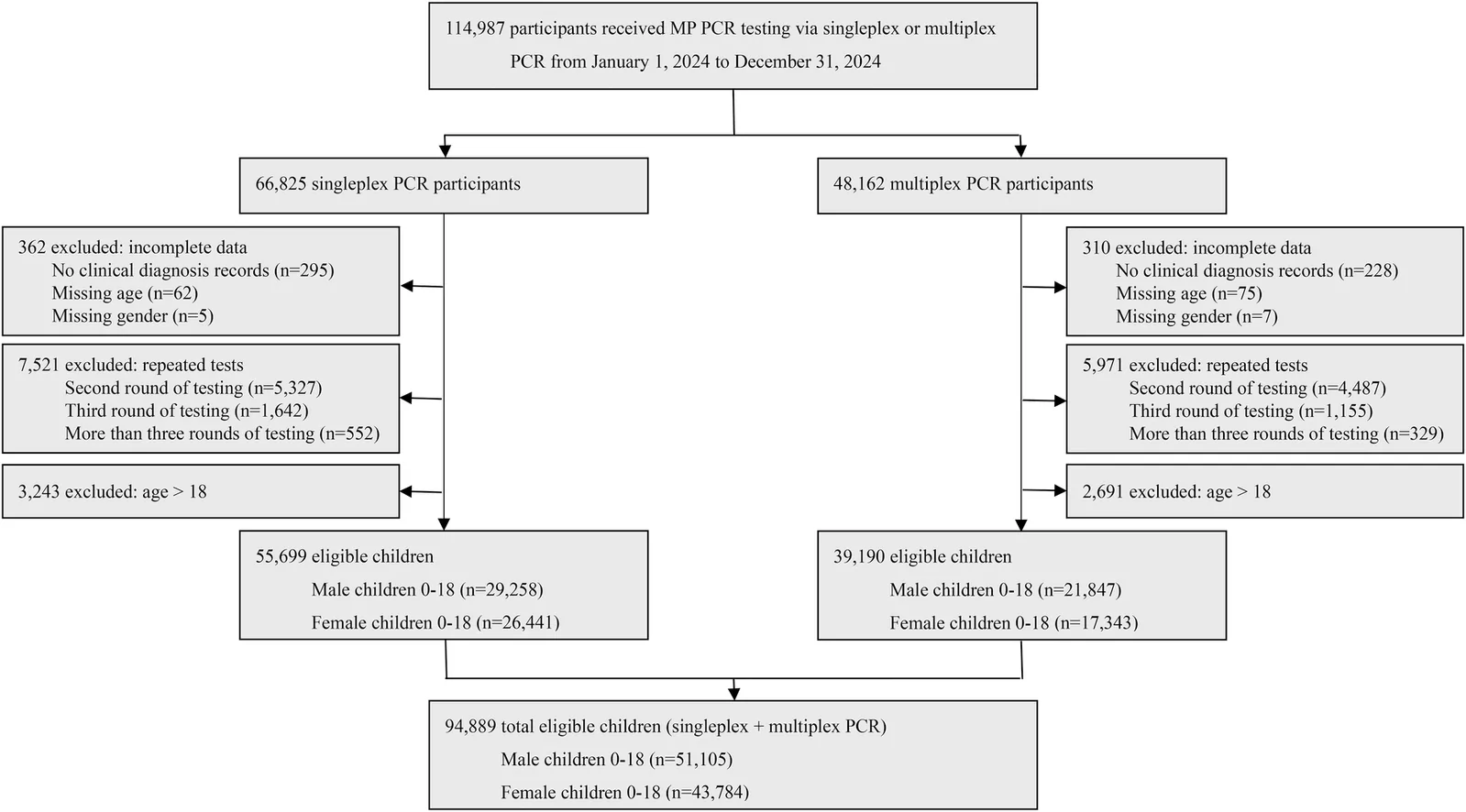
Flowchart of participant screening stratified by singleplex and multiplex PCR for eligible children aged 0–18 years.

### Comparison of MP positivity rates between male and female children detected by singleplex or multiplex PCR

Among all 94,889 included children, the overall MP positivity rate was 12.3% (95% CI: 12.12–12.54). The MP positivity rates were 12.2% (95% CI: 11.94–12.50) in males, and 12.5% (95% CI: 12.15–12.77) in females, respectively, with no statistically significant sex difference (*p* = 0.256). In stratified analyses by PCR platform, the MP positivity rates were 12.8% (95% CI: 12.47–13.03) for singleplex PCR, and 11.73% (95% CI: 11.41–12.05) for multiplex PCR, respectively. Detailed comparisons of MP positivity rates between males and females, stratified by singleplex or multiplex PCR, are presented in Table 1.

**Table 1:** Comparisons of MP positivity rates between male and female children detected by singleplex or multiplex PCR (%, n/N).

	Singleplex PCR			Multiplex PCR			Total		
	n/N	positivity rates	95% CI	n/N	positivity rates	95% CI	n/N	positivity rates	95% CI
Total	7,104/55,699	12.8	12.47–13.03	4,596/39,190	11.7	11.41–12.05	11,700/94,889	12.3	12.12–12.54
Males	3,742/29,258	12.8	12.41–13.17	2,502/21,847	11.5	11.03–11.87	6,244/51,105	12.2	11.94–12.50
Females	3,362/26,441	12.7	12.32–13.12	2,094/17,343	12.1	11.59–12.55	5,456/43,784	12.5	12.15–12.77
*χ* ^2^		0.07			3.61			1.29	
*p*-value		0.792			0.057			0.256	

### Comparisons of MP positivity rates between inpatient and outpatient/emergency children, and between males and females across different clinical diagnostic groups

Across five clinical diagnostic groups, MP positivity rate was the highest in the ALRTIs group (13.9%, 95% CI: 13.62–14.16), followed by the SRLT group (12.8%, 95% CI: 11.70–13.82); the positivity rate in these two groups was significantly higher than that in the CRDs group (8.8%, 95% CI: 5.92–11.72), AURTIs group (6.6%, 95% CI: 6.15–7.01), and NRDs group (4.8%, 95% CI: 4.23–5.35) (*χ*^2^ = 840.99, *p* < 0.001). In terms of healthcare settings, varying MP positivity patterns between inpatient and outpatient/emergency children were observed across diagnostic subgroups, with significant differences found in most groups. Detailed stratified comparisons are illustrated in Figure 2. In terms of sex, among the five different clinical diagnostic groups, no statistically significant differences in MP positivity rate were observed between male and female children in each group (all *p* > 0.05). Corresponding stratified comparisons are shown in Figure 3.

**Figure 2:**
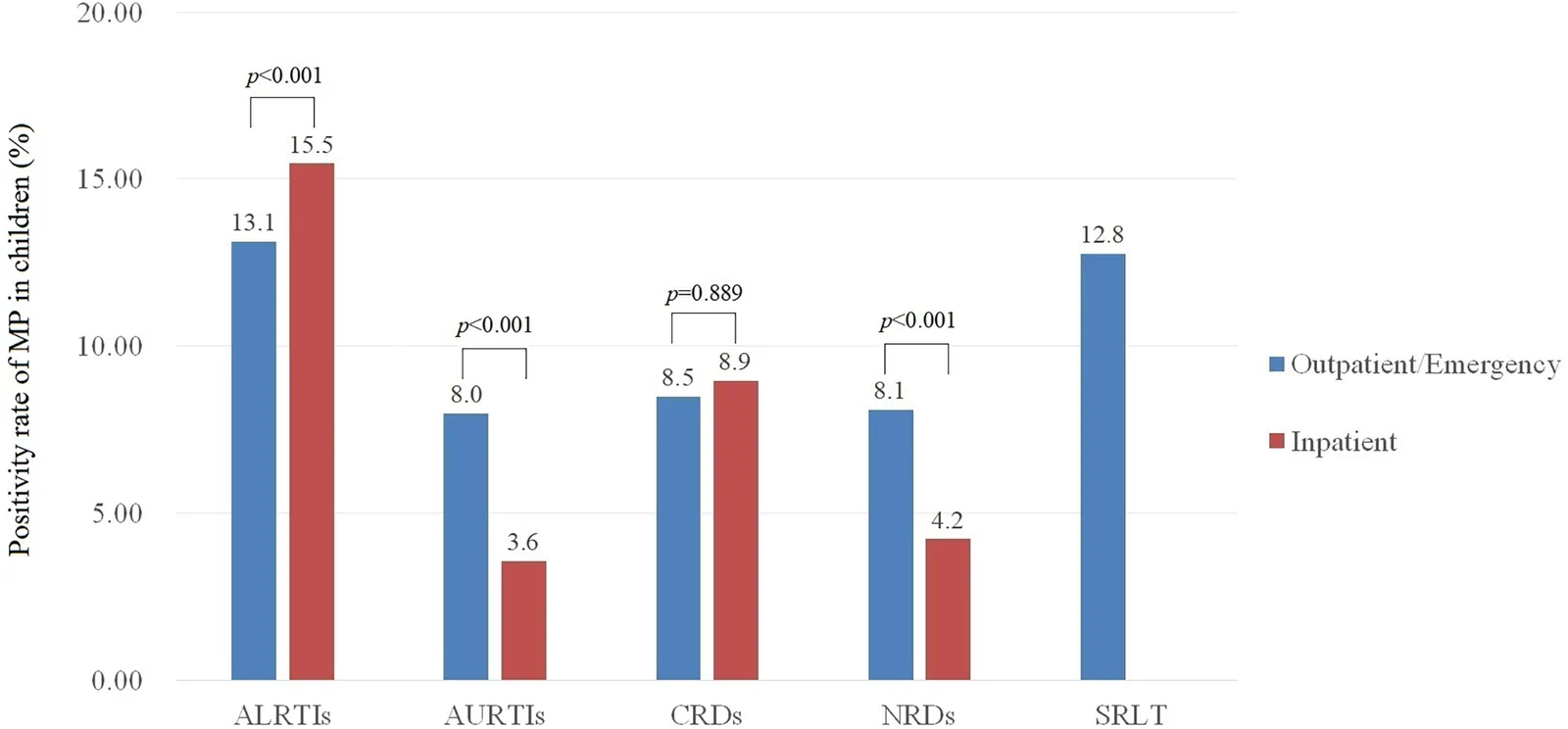
Comparisons of MP positivity rates between inpatient and outpatient/emergency children across different clinical diagnostic groups. The MP positivity rates (%, n/N) for each diagnostic subgroup are presented as follows. *n* = MP-positive children, *N* = total tested children. ALRTIs: overall 13.9% (10,104/72,752), 95% CI: 13.62–14.16; outpatient/emergency 13.1% (6,463/49,205), 95% CI: 12.83–13.43; inpatient 15.5% (3,641/23,547), 95% CI: 15.00–15.92. AURTIs: overall 6.6% (829/12,601), 95% CI: 6.15–7.01; outpatient/emergency 8.0% (687/8,609), 95% CI: 7.41–8.55; inpatient 3.6% (142/3,992), 95% CI: 2.98–4.14. CRDs: overall 8.8% (32/363), 95% CI: 5.92–11.72; outpatient/emergency 8.5% (9/106), 95% CI: 3.19–13.79; inpatient 8.9% (23/257), 95% CI: 5.46–12.44. NRDs: overall 4.8% (262/5,466), 95% CI: 4.23–5.35; outpatient/emergency 8.1% (65/802), 95% CI: 6.22–9.98; inpatient 4.2% (197/4,664), 95% CI: 3.64–4.80. SRLT: only outpatient/emergency cases 12.8% (473/3,707), 95% CI: 11.70–13.82.

**Figure 3:**
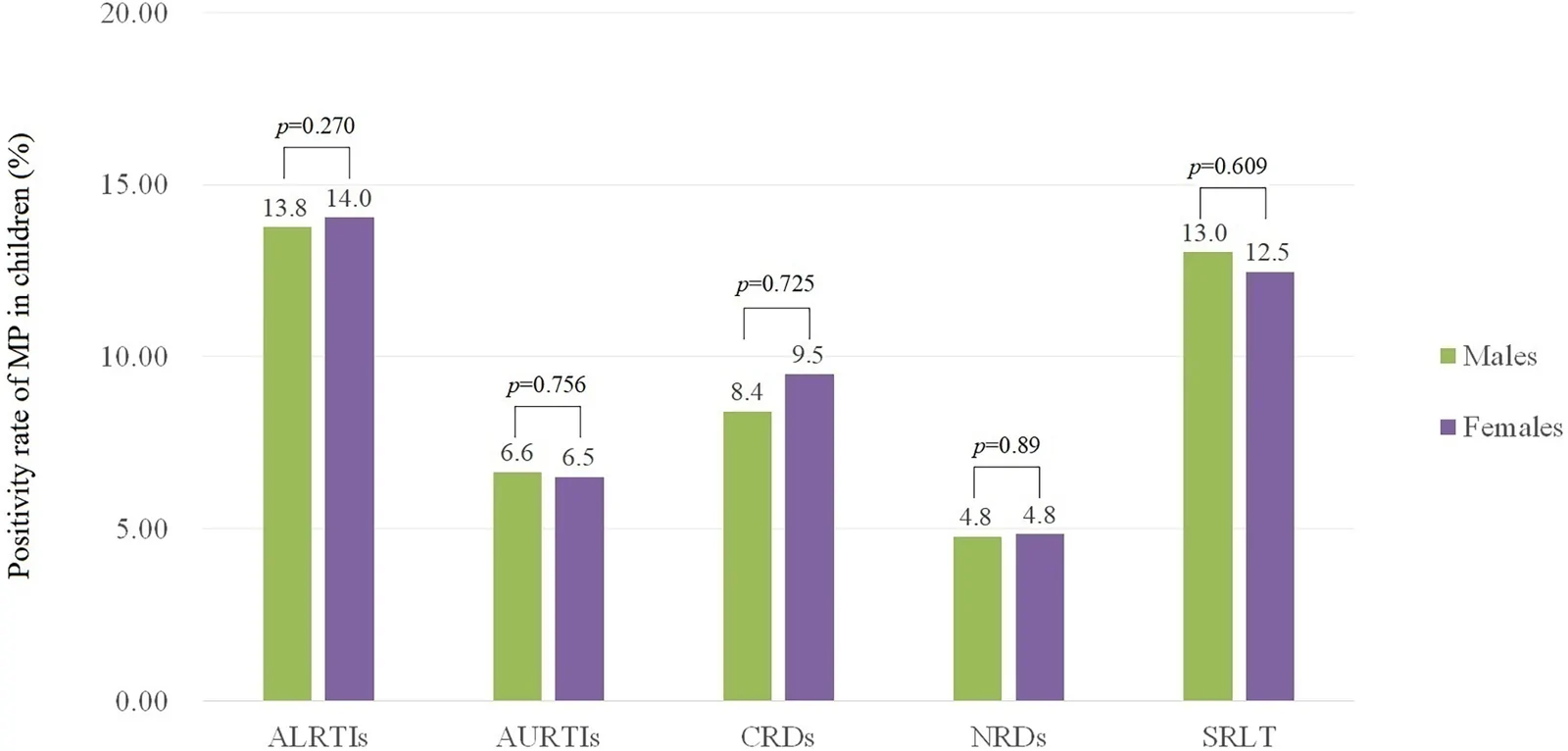
Comparisons of MP positivity rates between male and female children across different clinical diagnostic groups. The MP positivity rates (&percnt;, n/N) for each diagnostic subgroup are presented as follows. <italic>n</italic> &equals; MP-positive children, <italic>N</italic> &equals; total tested children. ALRTIs: overall 13.9&percnt; (10,104/72,752), 95&percnt; CI: 13.62–14.16; males 13.8&percnt; (5,371/39,042), 95&percnt; CI: 13.42–14.10; females 14.0&percnt; (4,733/33,710), 95&percnt; CI: 13.67–14.41. AURTIs: overall 6.6&percnt; (829/12,601), 95&percnt; CI: 6.15–7.01; males 6.6&percnt; (457/6,881), 95&percnt; CI: 6.06–7.22; females 6.5&percnt; (372/5,720), 95&percnt; CI: 5.86–7.14. CRDs: overall 8.8% (32/363), 95&percnt; CI: 5.92–11.72; males 8.4&percnt; (19/226), 95&percnt; CI: 4.77–12.05; females 9.5&percnt; (13/137), 95&percnt; CI: 4.56–14.42. NRDs: overall 4.8&percnt; (262/5,466), 95&percnt; CI: 4.23–5.35; males 4.8&percnt; (143/3,006), 95&percnt; CI: 3.99–5.53; females 4.8&percnt; (119/2,460), 95&percnt; CI: 3.99–5.69. SRLT: overall 12.8&percnt; (473/3,707), 95% CI: 11.70–13.82; males 13.0&percnt; (254/1,950), 95&percnt; CI: 11.54–14.52; females 12.5&percnt; (219/1,757), 95&percnt; CI: 10.92–14.00.

### Comparison of MP positivity rates in children of different age groups across different clinical diagnostic groups

Across the five clinical diagnostic groups, significant inter-age differences in MP positivity rates were observed in ALRTIs, AURTIs, NRDs, and SRLT (*χ*^2^-values ranged from 25.80 to 551.67; all *p* < 0.001). Within these four diagnostic subgroups, school-aged children demonstrated the highest MP positivity rates, followed by preschool children and adolescents. However, no statistically significant differences in MP positivity rates were found across age groups for children with CRDs (*p* = 0.604). Full stratified comparisons of MP positivity rates by age and clinical diagnosis, including positivity estimates, 95% CIs and pairwise significance results, are presented in Table 2.

**Table 2:** Comparisons of MP positivity rates in children of different age groups across different clinical diagnostic groups (%, n/N).

Age groups	ALRTIs		AURTIs		CRDs		NRDs		SRLT		*χ* ^2^	*p*-value
	Positivity rates	95% CI	Positivity rates	95% CI	Positivity rates	95% CI	Positivity rates	95% CI	Positivity rates	95% CI		
Newborns	1.4 (17/1,230)	0.74–2.02	5.4 (2/37)	0.00–12.75	0.0 (0/1)	0.00–0.00	0.5 (1/201)	0.00–1.48	/ (0/0)	/	43.30	<0.001
Infants	4.9 (383/7,803)^a^	4.43–5.39	1.9 (17/899)	1.00–2.78	12.5 (1/8)	0.00–35.42	2.5 (15/602)	1.25–3.73	7.0 (8/114)	2.33–11.71	25.80	<0.001
Toddlers	8.9 (1,179/13,241)^a^^,^^b^	8.42–9.39	3.0 (83/2,757)	2.37–3.65	9.2 (7/76)	2.71–15.71	3.6 (39/1,081)^a^	2.50–4.72	6.2 (40/646)	4.33–8.05	141.85	<0.001
Preschool children	12.8 (3,616/28,261)^a^^,^^b^^,^^c^	12.41–13.19	6.1 (263/4,329)^b^^,^^c^	5.37–6.79	5.9 (8/135)	1.95–9.91	5.1 (78/1,537)^a^^,^^b^	3.97–6.17	8.9 (132/1,476)^c^	7.48–10.40	248.32	<0.001
School-aged children	22.9 (4,689/20,453)^a^^,^^b^^,^^c^^,^^d^	22.35–23.51	10.4 (443/4,244)^c^^,^^d^	9.52–11.36	11.7 (16/137)	6.30–17.60	6.7 (116/1,726)^a^^,^^b^^,^^c^^,^^d^	5.54–7.90	21.4 (289/1,352)^b^^,^^c^^,^^d^	19.19–23.57	551.67	<0.001
Adolescents	12.5 (220/1,764)^a^^,^^b^^,^^c^^,^^f^	10.93–14.01	6.3 (21/335)^b^^,^^c^^,^^f^	3.67–8.87	0.0 (0/6)	0.00–0.00	4.1 (13/319)^a^	1.91–6.25	3.4 (4/119)^e^^,^^f^	0.12–6.60	35.57	<0.001
*χ* ^2^	2,390.04		194.04		3.63		33.12		147.33			
*p*-value	<0.001		<0.001		0.604		<0.001		<0.001			

n refers to the number of MP-positive cases, and N refers to the total number of observed participants in the group.

a Significantly increased compared with the newborns.

b Significantly increased compared with the infants.

c Significantly increased compared with the toddlers.

d Significantly increased compared with the preschool children.

e Significantly decreased compared with the preschool children.

f Significantly decreased compared with the school-aged children.

### Comparison of MP positivity rates in different seasons across different clinical diagnostic groups

Within 2024, across the five clinical diagnostic groups, significant differences in MP positivity rates were observed within each season group (spring, summer, autumn, or winter) (*χ*^2^-values ranged from 98.01 to 287.26; all *p* < 0.001). The MP positivity rates in the ALRTIs, AURTIs, NRDs, and SRLT groups were the highest in winter (19.2%, 95% CI: 18.72–19.68; 11.8%, 95% CI: 10.73–12.81; 9.5%, 95% CI: 7.95–10.97; and 17.5%, 95% CI: 15.80–19.14; respectively), followed by spring. The rates in winter and spring were significantly higher than those in summer and autumn for these four groups (all *p* < 0.001). Although no statistically significant seasonal differences in MP positivity rates were observed in the CRDs group (*p* = 0.057), the rates were still higher in winter and spring (16.4%, 95% CI: 7.10–25.68 and 10.4%, 95% CI: 4.57–16.19) and lower in summer and autumn (6.9%, 95% CI: 1.57–12.23; and 4.6%, 95% CI: 0.66–8.52). The comparisons of MP positivity rates in different seasons across different clinical diagnostic groups are reported in Table 3.

**Table 3:** Comparisons of MP positivity rates in different seasons across different clinical diagnostic groups (%, n/N).

Seasons	ALRTIs		AURTIs		CRDs		NRDs		SRLT		*χ* ^2^	*p*-value
	Positivity rates	95% CI	Positivity rates	95% CI	Positivity rates	95% CI	Positivity rates	95% CI	Positivity rates	95% CI		
Spring (Mar.–May.)	13.2 (2,421/18,411)	12.66–13.64	6.2 (154/2,470)	5.28–7.18	10.4 (11/106)	4.57–16.19	4.4 (60/1,367)	3.30–5.48	9.6 (67/698)	7.41–11.79	181.71	<0.001
Summer (Jun.–Aug.)	12.2 (1,850/15,117)^a^	11.72–12.76	4.5 (172/3,830)^a^	3.83–5.15	6.9 (6/87)	1.57–12.23	3.6 (52/1,446)	2.64–4.56	6.6 (43/652)^a^	4.69–8.51	287.26	<0.001
Autumn (Sep.–Nov.)	6.0 (771/12,858)^a^^,^^c^	5.59–6.41	2.5 (65/2,581)^a^^,^^c^	1.92–3.12	4.6 (5/109)	0.66–8.52	1.1 (13/1,205)^a^^,^^c^	0.50–1.66	4.3 (16/371)^a^	2.24–6.38	98.01	<0.001
Winter (Dec.–Feb.)	19.2 (5,062/26,366)^b^^,^^d^^,^^e^	18.72–19.68	11.8 (438/3,720)^b^^,^^d^^,^^e^	10.73–12.81	16.4 (10/61)	7.10–25.68	9.5 (137/1,448)^b^^,^^d^^,^^e^	7.95–10.97	17.5 (347/1,986)^b^^,^^d^^,^^e^	15.80–19.14	196.14	<0.001
*χ* ^2^	1,334.24		260.25		7.50		110.60		91.93			
*p*-value	<0.001		<0.001		0.057		<0.001		<0.001			

n refers to the number of MP-positive cases, and N refers to the total number of observed participants in the group.

a Significantly decreased compared with the spring.

b Significantly increased compared with the summer.

c Significantly decreased compared with the summer.

d Significantly increased compared with the autumn.

e Significantly increased compared with the autumn.

### Comparison of MP monthly positivity rates from January to December across different clinical diagnostic groups

Significant temporal variations in monthly MP positivity rates were detected throughout 2024 within the ALRTIs, AURTIs, NRDs, and SRLT groups (*χ*^2^ ranging from 211.10 to 3733.99, all *p* < 0.001). Peak MP positivity occurred in January and February across these four subgroups, followed by an overall declining trend with mild fluctuations, with trough values observed in summer and autumn. Although no statistically significant temporal differences in monthly MP positivity rates were observed in the CRDs group from January to December 2024 (*χ*^2^ = 19.10, *p* = 0.059), MP positivity tended to be higher in early months (January–February) and reached 0% in August and December. Full monthly trends, positivity estimates and corresponding 95% confidence intervals are illustrated in Figure 4.

**Figure 4:**
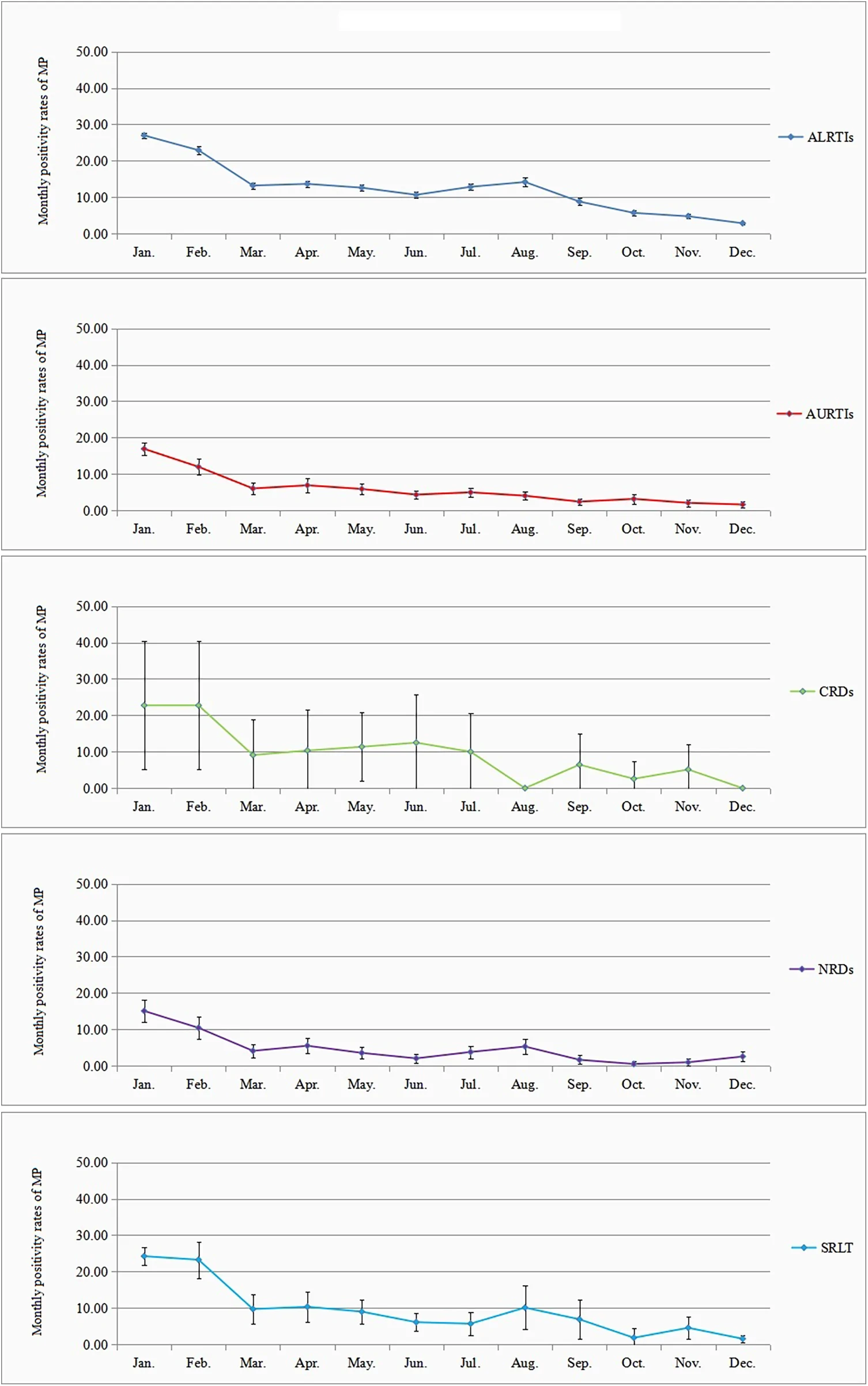
Monthly positivity rates of MP in children across different clinical diagnostic groups (Jan. to Dec. 2024, %). *n* = MP-positive children, *N* = total tested children. Each plotted monthly data point represents the MP positivity rate calculated from monthly n/N within each diagnostic subgroup; vertical error bars denote 95% Wilson CI for monthly positivity estimates. The aggregated annual MP positivity rates (%, n/N) and their 95% CI for each diagnostic category are summarized as follows: ALRTIs: overall 13.9% (10,104/72,752), 95% CI: 13.62–14.16; AURTIs: overall 6.6% (829/12,601), 95% CI: 6.15–7.01; CRDs: overall 8.8% (32/363), 95% CI: 5.92–11.72; NRDs: overall 4.8% (262/5,466), 95% CI: 4.23–5.35; SRLT: only outpatient/emergency cases 12.8% (473/3,707), 95% CI: 11.70–13.82.

### Associations between MP positivity and variables in child participants: univariate logistic regression analysis

Univariate logistic regression analysis revealed that, compared with newborns, MP positivity was significantly higher in infants, toddlers, preschool children, school-aged children, and adolescents, with ORs ranging from 3.41 to 17.99 (all *p* < 0.001). Compared with spring, MP positivity was significantly lower in summer and autumn (OR: 0.84 and 0.40, respectively) but higher in winter (OR: 1.63). All associations were statistically significant (*p* < 0.001). Compared with the ALRTIs group, MP positivity was significantly lower in the AURTIs, CRDs, and NRDs groups (OR: 0.44, 0.60, and 0.31, respectively; all *p* < 0.05). For the SRLT group, the univariate analysis showed a lower odds of a positive MP test compared with the ALRTIs group (OR: 0.91, 95% CI: 0.82–1.00, *p* = 0.052). In terms of PCR detection methods, children tested by multiplex PCR exhibited a significantly lower MP positivity risk relative to those tested by singleplex PCR (OR: 0.91, 95% CI: 0.87–0.95, *p* < 0.001). Conversely, no statistically significant associations were found between MP positivity and healthcare settings (inpatient vs. outpatient/emergency, OR: 1.00, *p* = 0.990) or sex (female vs. male, OR: 1.02, *p* = 0.256). The associations between MP positivity and variables (healthcare settings, sex, age, seasons, clinical diagnoses, PCR assay type) in children are reported in Table 4.

**Table 4:** Univariate logistic regression analysis of associations between MP positivity and variables (healthcare setting, sex, age, season, clinical diagnosis, PCR assay type) in child participants.

Variables	Odds ratio	95% CI	Wald	*p*-value
Healthcare settings				
Outpatient/emergency	1	–	–	–
Inpatient	1.00	0.96–1.04	0.00	0.990
Sex				
Males	1	–	–	–
Female	1.02	0.98–1.06	1.29	0.256
PCR assay type				
Singleplex	1	–	–	–
Multiplex	0.91	0.87–0.95	22.42	<0.001
Age groups				
Newborns	1	–	–	–
Infants	3.41	2.17–5.36	28.34	<0.001
Toddlers	5.94	3.81–9.26	61.60	<0.001
Preschool children	9.38	6.03–14.60	98.34	<0.001
School-aged children	17.99	11.56–28.00	164.04	<0.001
Adolescents	8.18	5.17–12.95	80.31	<0.001
Seasons				
Spring	1	–	–	–
Summer	0.84	0.79–0.89	33.50	<0.001
Autumn	0.40	0.37–0.43	511.93	<0.001
Winter	1.63	1.55–1.71	383.42	<0.001
Clinical diagnoses				
ALRTIs	1	–	–	–
AURTIs	0.44	0.41–0.47	488.34	<0.001
CRDs	0.60	0.42–0.86	7.62	0.006
NRDs	0.31	0.28–0.35	328.68	<0.001
SRLT	0.91	0.82–1.00	3.77	0.052

### Associations between MP positivity and variables in child participants: multivariable logistic regression analysis (stepwise forward Wald)

After adjustment for potential confounders, multivariable logistic regression analysis revealed that MP positivity was independently associated with healthcare settings (inpatient vs. outpatient/emergency, OR: 1.24, 95% CI: 1.15–1.35, *p* < 0.001), as well as PCR assay type (multiplex vs. singleplex, OR: 2.45, 95% CI: 2.25–2.67, *p* < 0.001). Compared with newborns, MP positivity was independently and positively associated with all other age groups (infants, toddlers, preschool children, school-aged children, and adolescents), with significantly higher ORs ranging from 3.89 to 31.94 (all *p* < 0.001). The strongest association was observed in school-aged children (OR: 31.94, 95% CI: 20.45–49.88). Compared with spring, MP positivity was independently and inversely associated with summer (OR: 0.85, 95% CI: 0.80–0.91, *p* < 0.001) and autumn (OR: 0.68, 95% CI: 0.63–0.74, *p* < 0.001), but positively associated with winter (OR: 1.98, 95% CI: 1.89–2.09, *p* < 0.001). Compared with the ALRTIs group, MP positivity was independently and inversely associated with the AURTIs, CRDs, NRDs, and SRLT groups (ORs ranging from 0.13 to 0.28; all *p* < 0.001). The lowest OR was observed in the NRDs group (OR: 0.13, 95% CI: 0.11–0.15), while the highest OR was found in the AURTIs group (OR: 0.28, 95% CI: 0.26–0.31). The associations between MP positivity and variables (healthcare settings, sex, age, seasons, clinical diagnoses, PCR assay type) in children were analysed by multivariable logistic regression, and the results are reported in Table 5.

**Table 5:** Multivariable logistic regression analysis of associations between MP positivity and variables in 94,889 children aged 0–18 (stepwise forward Wald).

Variables	Odds ratio	95% CI	Wald	*p*-value
Healthcare settings				
Outpatient/emergency	1	–	–	–
Inpatient	1.24	1.15–1.35	27.26	<0.001
PCR assay type				
Singleplex	1	–	–	–
Multiplex	2.45	2.25–2.67	409.89	<0.001
Age groups				
Newborns	1	–	–	–
Infants	3.89	2.47–6.12	34.36	<0.001
Toddlers	9.39	6.00–14.69	96.23	<0.001
Preschool children	16.78	10.75–26.20	153.85	<0.001
School-aged children	31.94	20.45–49.88	232.00	<0.001
Adolescents	13.86	8.72–22.04	123.53	<0.001
Seasons				
Spring	1	–	–	–
Summer	0.85	0.80–0.91	27.36	<0.001
Autumn	0.68	0.63–0.74	79.17	<0.001
Winter	1.98	1.89–2.09	669.64	<0.001
Clinical diagnoses				
ALRTIs	1	–	–	–
AURTIs	0.28	0.26–0.31	825.97	<0.001
CRDs	0.18	0.10–0.30	40.02	<0.001
NRDs	0.13	0.11–0.15	741.71	<0.001
SRLT	0.16	0.13–0.19	486.54	<0.001

In order to systematically explore factors associated with the outcome variable (MP positivity), six variables including healthcare settings, sex, age, seasons, clinical diagnoses, and PCR assay type were directly included in the multivariable logistic regression analysis. Among these variables, sex was finally excluded from the MP models (method: multivariable logistic regression analysis using the Stepwise Forward Wald approach).

## Discussion

In the present retrospective analysis of 94,889 children undergoing MP testing in 2024, the overall MP positivity rate was 12.3%. Positivity was significantly higher among inpatients vs. outpatients/emergency children within the ALRTIs group (15.5% vs. 13.1%, *p* < 0.001), but lower among inpatients within the AURTIs (3.6% vs. 8.0%) and NRDs (4.2% vs. 8.1%) groups (both *p* < 0.001). Multivariable logistic regression analysis revealed that MP positivity was independently associated with the healthcare setting (inpatient vs. outpatient/emergency, OR: 1.24, 95% CI: 1.15–1.35, *p* < 0.001), while sex was excluded from the final multivariable models. This discrepancy was primarily driven by the disproportionate concentration of inpatients in the ALRTIs category—the group with the highest MP positivity—consistent with confounding by clinical diagnostic grouping.

We further observed divergent MP positivity patterns stratified by PCR detection platform. Multivariable regression indicated that multiplex PCR was independently associated with higher odds of a positive MP test compared with singleplex PCR (OR: 2.45, 95% CI: 2.25–2.67, *p* < 0.001). This adjusted result contrasted with the crude positivity trend, consistent with confounding driven by differences in clinical testing indications. Singleplex testing is typically requested with high pre-test probability of MP infection, while multiplex panels are deployed for children with undifferentiated respiratory symptoms. Stratified analysis revealed a borderline significant sex difference in MP positivity within the multiplex PCR subgroup, whereas no sex disparity was detected in the singleplex subgroup.

Previous studies have reported substantial variation in MP positivity across regions and periods. Roh EJ et al. (23) reported that the overall MP positivity rate among inpatients with CAP in South Korea (2018–2020) were 41.3%, and Wang F et al. (24) found that MP positivity in inpatients (27.2%–30.1%) were significantly higher than in outpatient/emergency cases (7.8%–10.1%) in Northeast China and Inner Mongolia (2017–2023). More recently, a Danish study by Nilsson SS et al. (25) reported an MP positivity rate of 18.9% in 2024. The MP positivity rate among inpatients with ALRTIs in our study (15.5%) was lower than that reported by Roh EJ et al. and Wang F et al. This discrepancy may be attributed to our broader definition of ALRTIs, which included milder conditions such as acute tracheitis and acute bronchitis, whereas some previous studies focused solely on CAP. Other potential factors include regional and temporal variations in MP epidemiology, and differences in detection methods. Consistent with many prior epidemiological studies, we detected no sex-based differences in MP positivity.

In addition, our findings across five clinical diagnostic groups indicated that the MP positivity rate was the highest in the ALRTIs group (13.9%), followed by the SRLT group (12.8%). Positivity rates in these two groups were significantly higher than those in the CRDs group (8.8%), AURTIs group (6.6%), and NRDs group (4.8%) (*p* < 0.001). Multivariable logistic regression analysis revealed that, compared with the ALRTIs group, the odds of a positive MP test were independently lower in the AURTIs, CRDs, NRDs, and SRLT groups (ORs ranging from 0.13 to 0.28; all *p* < 0.001); the NRDs group exhibited the lowest OR (OR: 0.13, 95% CI: 0.11–0.15), whereas the AURTIs group had the highest OR (OR: 0.28, 95% CI: 0.26–0.31). This pattern is consistent with the recognized tropism of MP for lower respiratory tract epithelium, although the heterogeneity of conditions within the ALRTIs category—including pneumonia, bronchitis, bronchiolitis, and tracheitis—limits the specificity of this interpretation. The relatively high MP positivity rate in the SRLT group may stem from proactive testing behavior: parents often request MP testing for children with mild respiratory symptoms to rule out infection. However, this group is subject to substantial self-selection bias, as parents who proactively requested testing may differ systematically from those in clinician-ordered groups in terms of symptoms, health-seeking behavior, or awareness of local MP circulation.

Numerous epidemiological studies have reported age-based variation in MP PCR positivity among children (26–28). Our findings revealed that among children with both ALRTIs and AURTIs, MP positivity was significantly higher in school-aged, preschool, and adolescent children than in toddlers, infants, and newborns (*p* < 0.001). Multivariable logistic regression analysis confirmed that age remained an independent predictor after adjusting for other covariates, with school-aged children showing the strongest association when newborns were used as the reference category (OR: 31.94, 95% CI: 20.45–49.88, *p* < 0.001).

Our age-related patterns are consistent with findings from Miao Y et al. (29) and Qiu W et al. (30), both of which reported the highest MP positivity rates in school-aged children across multiple years. Notably, our study extends these observations by demonstrating that school-aged children aged 6–12 years had higher MP positivity than adolescents aged 12–18 years—a finding consistent across all diagnostic subgroups and robust after multivariable adjustment. However, the markedly elevated odds of a positive MP test among school-aged children should be interpreted cautiously. These estimates were generated with newborns (who demonstrated very low MP positivity) as the reference group and may overestimate the magnitude of age-associated differences in absolute risk. The consistent age gradient across diagnostic groups highlights that age is an important consideration for interpreting MP test results in routine clinical practice.

Studies have shown that accumulated epidemiological surveillance data indicate temporal variation in MP PCR positivity across geographical regions (31, 32). Earlier investigations from different regions have documented diverse temporal patterns of MP positivity across China. Studies from northern China (Beijing, 2016–2021) reported higher MP positivity rates during summer and winter, while a study from Urumqi (2023–2024) documented sustained high MP positivity from autumn through spring (33, 34). In southern China, studies from Wuhan (2018–2024) identified autumn-dominant peaks, and a study from Shanghai (2022–2023) recorded a rapid increase starting in late summer 2023 (35, 36).

In this study, within each season, MP positivity rates differed significantly across the five clinical diagnostic groups (all *p* < 0.001). When comparing across seasons within each diagnostic group, for the ALRTIs, AURTIs, NRDs, and SRLT groups, the highest MP positivity rates were observed in winter, driven by elevated levels in January and February, with significantly lower rates in summer and autumn (all *p* < 0.001). In the CRDs group, although no statistically significant seasonal difference was observed across the four seasons (*χ*^2^ = 7.50, *p* = 0.057; Table 3), the positivity rates remained higher in winter and spring and lower in summer and autumn. Multivariable logistic regression analysis showed that, compared with spring, the odds of a positive MP test were independently lower in summer (OR: 0.85, 95% CI: 0.80–0.91, *p* < 0.001) and autumn (OR: 0.68, 95% CI: 0.63–0.74, *p* < 0.001), while the odds of a positive MP test were higher in winter (OR: 1.98, 95% CI: 1.89–2.09, *p* < 0.001).

Furthermore, monthly MP positivity rates in the ALRTIs, AURTIs, NRDs, and SRLT groups varied significantly from January to December 2024 (all *p* < 0.001), with the highest rates observed in January and February. After February, the monthly rates generally declined with minor fluctuations, rather than following a strictly monotonic decreasing trend. The January–February peak observed in our analysis should be interpreted as a pattern identified within a single calendar year. These findings cannot confirm stable long-term seasonal periodicity, and multi-year surveillance data are required to verify whether this temporal pattern recurs consistently across years.

The present study possesses several notable strengths, including the large sample size of over 90,000 children, PCR-based detection, inclusion of both inpatient and outpatient/emergency settings, and detailed age and temporal stratifications. Testing was performed using both singleplex and multiplex PCR platforms, with assay type incorporated as a covariate in regression models. Simultaneous adjustment for multiple demographic and clinical confounders allowed assessment of independent correlates of MP positivity. These strengths should be weighed against the limitations of the observational design and sampling strategy outlined below.

### Study limitations

Several limitations should be acknowledged. The single-center design limits generalizability, and the retrospective nature may introduce bias from incomplete records and unmeasured confounders (e.g., socioeconomic status, household crowding, prior infections). Grouping patients into broad diagnostic categories, particularly the heterogeneous ALRTI group, may obscure condition-specific MP positivity patterns. Data on antimicrobial resistance and co-infections were not collected. Newborns served as the reference group in age-stratified analyses, which may inflate odds ratios for other age subgroups. Seasonal findings are based on a single year and require multi-year validation; the small CRDs subgroup (*n* = 363) further limits precision. Statistical limitations include the absence of formal multicollinearity diagnostics and the potential overestimation of associations using logistic regression (ORs) instead of modified Poisson regression. Future multicenter studies with prospective design and comprehensive data collection are needed to address these gaps.

## Conclusions

This retrospective analysis of 94,889 children receiving MP PCR testing in Chengdu during 2024 demonstrated an overall MP PCR positivity rate of 12.3%, with no sex-based difference. MP positivity varied substantially across clinical diagnostic groups, being highest among children with ALRTIs, followed by the SRLT group; rates in these two groups were markedly higher than those observed in children with CRDs, AURTIs and NRDs. Distinct age-related and seasonal epidemiological patterns were identified: positivity peaked among school-aged children (6–12 years) and reached its highest level in winter, with January and February as the peak months. Stratified analysis revealed divergent patterns of MP positivity by healthcare setting across diagnostic subgroups: within the ALRTIs group, inpatients exhibited higher MP positivity than outpatient/emergency children, whereas the opposite pattern was observed for children with AURTIs. Multivariable logistic regression confirmed that healthcare setting and PCR assay type were independently associated with a positive MP test after adjustment for age, season, and clinical diagnosis. These findings characterize local one-year epidemiological features of MP PCR positivity among tested children. Our results can support targeted clinical testing strategies and regional infectious disease surveillance, athough multi-year continuous surveillance is needed to validate whether the observed seasonal and age-related patterns persist over longer periods.

## Data Availability

The datasets used and analyzed during the current study are available from the corresponding author on reasonable request. Requests to access these datasets should be directed to Chenggui Liu, lablcg@126.com or Weijun He, 15928155110@163.com.
